# Metabolite Profiling of Preneoplastic and Neoplastic Lesions of Oral Cavity Tissue Samples Revealed a Biomarker Pattern

**DOI:** 10.1038/srep38985

**Published:** 2016-12-13

**Authors:** Syed Ghulam Musharraf, Najia Shahid, Syed Muhammad Ali Naqvi, Mahwish Saleem, Amna Jabbar Siddiqui, Anwar Ali

**Affiliations:** 1 Dr. Panjwani Center for Molecular Medicine and Drug Research, International Center for Chemical and Biological Sciences, University of Karachi, Karachi-75270, Pakistan; 2 H.E.J. Research Institute of Chemistry, International Center for Chemical and Biological Sciences, University of Karachi, Karachi-75270, Pakistan; 3 Dr. Ishrat-ul-Ebad Institute of Oral Health Sciences, DOW University of Health Sciences, Karachi-74200, Pakistan

## Abstract

Oral cancer is a major health challenge in the Indian subcontinent and a dreadful form of cancers worldwide. The current study is focused on the identification of distinguished metabolites of oral cancer tissue samples in comparison with precancerous and control tissue samples using gas chromatography coupled with triple quadrupole tandem mass spectrometry and chemometric analyses. Metabolites obtained were identified through National Institute of Standards and Technology (NIST) mass spectral (Wiley registry) library. Mass Profiler Professional (MPP) software was used for the alignment and for all the statistical analysis. 31 compounds out of 735 found distinguishing among oral cancer, precancerous and control group samples using *p-*value ≤ 0.05. Partial Least Square Discriminant Analysis (PLSDA) model was generated using statistically significant metabolites gave an overall accuracy of 90.2%. Down-regulated amino acid levels appear to be the result of enhanced energy metabolism or up-regulation of the appropriate biosynthetic pathways, and required cell proliferation in cancer tissues. These results suggest that tissue metabolic profiles have great potential in detecting oral cancer and may aid in understanding its underlying mechanisms.

Oral cancer is a continuing global challenge ranking sixth most common malignant tumor worldwide^1^. IARC (International Agency for Research on Cancer) in a latest report on oral cancer presented a higher annual incidence of over 300,000 diagnosed cases, and the annual mortality of 145,000 death around the world^2^. GLOBOCAN 2012 present the most critical incidence of oral cancer by WHO South-East Asia region (SEARO) in India, Pakistan, Bangladesh, Sri Lanka and Taiwan^3^. In Pakistan it is the second commonest as per recent records of an established and well maintained cancer registry of Shaukat Khanum Memorial Cancer Hospital^4^.

More than 90% of oral cancers are oral squamous cell carcinoma (OSCC) which originates from pre-existing potentially malignant disorders or more often from normal appearing oral mucosal lining. Potentially malignant disorders such as oral sub mucous fibrosis (OSF) and oral leukoplakia (OLK) are early indicators of damage to oral mucosa with a transformation of 2–12% to frank malignancies^5^,^6^. Prevalence of OSF can be predominantly seen in South East Asia, more common in India and Pakistan, due to the chronic use of betel nuts and its products which cause mutagenic and genetotoxic effects. Malignant transformation rate of OSF was found to be in the range of 7–13% in a recent study, and has one of the highest rates amongst potentially malignant oral lesions and conditions^7^,^8^.

This is a globally accepted fact that early detection of cancer greatly increases the chances for successful treatment. Unfortunately a critical issue in the lack of prognostic improvement in oral cancer is the fact that a significant proportion of oral cancers initially are asymptomatic lesions and is not diagnosed or treated until they reach an advanced stage^5^. Visual detection of premalignant oral conditions and lesions has remained problematic throughout the world. They are highly heterogeneous lesions and not easily detected by naked eyes, resulted a high proportion of advanced stage OSCC with frequent relapse reported in recent years, showed a poor survival rate of patients with this devastating disease^9^.

It has been strongly proposed that development of effective clinical diagnostic aids and discovery of reliable biomarkers can allow early detection of OSCC or relapse which promise a definitive diagnosis of cancerous and precancerous oral lesions^10^,^11^. Metabolomic studies of various cancers tissue/plasma samples are not only helping in identifying therapeutic targets but also helping to detect diseases at a very early stage. Recently, a large scale study called European FP7 META cancer consortium project has utilized metabolomic approach to grade breast tumors^12^. However, and surprisingly, limited studies on metabolic profiling of oral cancers tissues have been conducted to date^13^,^14^. The current study proposed a tissue-based metabolomics profiling of potentially malignant oral disorders i.e. OSF and OSCC tissue specimens and its comparison with healthy control tissue samples which can initiate discovery of clinically useful metabolomics biomarkers used as a prognostic tool for early detection of oral cancer.

## Methods

### Sample Collection

Samples were collected from Dr. Ishrat-ul-Ebad Institute of Oral health Sciences, DOW University of Health Sciences, Karachi, Pakistan. Written informed content was obtained from all the subjects prior to biopsies according to the formatted consent forms and the study was approved from the Institutional Review Board of Dow University of Health Sciences, Karachi and Independent Ethic Committee (IEC), ICCBS, Karachi as well. Sample collection was carried out in accordance with relevant guidelines and regulations. 4–6 mm punch biopsies of total 51 samples were taken for the study, 15 samples of potentially malignant disorder of oral cavity that is of oral sub-mucous fibrosis (OSF), 21 of diagnosed OSCC patients and 15 from the healthy individuals (subject characteristics are shown in Supplementary Table 1). In control group, normal tissue punch biopsies have been taken during surgical extraction of lower third molar teeth. The oral mucosal punch is a rapid, simple, safe and inexpensive technique for obtaining a representative sample of most oral zones. All patients had not received any kind of therapy before sample collection and it was also taken into consideration that they were not in remission or relapse stage. All tissues were snap-freezed in liquid nitrogen immediately after surgery and preserved at −80 °C till sample preparation.

### Chemicals and Reagents

All solvents were of analytical grade. Methanol, choloroform and pyridine were purchased from Tedia (Tedia way, Fairfield, USA), while myristic-d_27_ acid was purchased from Sigma-Aldrich (St. Louis, MO, USA, respectively). BSTFA (N,O-Bis-(trimethylsilyl) trifluoroacetamide) and methoxylamine hydrochloric were purchased from Acros Organic (New Jersey, USA). Deionized water (Milli-Q) was used throughout the study (Millipore, Billerica, MA, USA).

### Sample Preparation

10 mg of tissue sample were transferred into 1.5 mL Eppendorf tube and homogenized for 2–3 minutes in 1000 μL of solvent mixture (MeOH:H_2_O:CHCl_3_ = 5:2:2). Then, 20 μL of myristic acid (2 mg/mL) as an internal standard was added and mixed well. The mixture was subsequently shaken at 1200 rpm at 4 °C for 2 hrs, then centrifuged at 14000 rpm for 5 minutes at 4 °C resultant clear supernatant was collected and dried in vacuum.

The dried extract of all the samples were derivatized subsequently by adding 80 μL methoxylamine hydrochloride in pyridine (15 μg/μL), vortexed and left for 1 hr at 30 °C. Then 80 μL BSTFA was added with 1% TCMS and placed at 35 °C for 1 hr to form trimethylsilyl (TMS) derivatives. The derivatized samples were centrifuged and analyzed within 24 hours.

### GC-MS Analyses

Derivatized samples were analyzed on a 7890 A gas chromatograph (Agilent technologies, USA) equipped with an Agilent Technology GC sampler 120 (PAL LHX-AG12) auto sampler and coupled to an Agilent 7000 Triple Quad system (Agilent technologies, USA). An HP-5MS 30 m–250 mm (i.d.) fused-silica capillary column (Agilent J&W Scientific, Folsom, CA, USA), chemically bonded with a 5% diphenyl 95% dimethylpolysiloxane cross-linked stationary phase (0.25 mm film thickness) was used. Helium was used as the carrier gas at 1.0 mL/min and the sample was injected in splitless mode. The injector and source temperatures were 250 °C. The oven temperature was initially maintained at 40 °C, and was then increased at 10 °C min to 300 °C and retained at 300 °C for 9 min. In post run, temperature was further increased to 305 °C, for remaining 1 min. Retention time was locked to myristic-d27 acid at 15.167 min. Electron ionization (EI) is used as an ionization source. Data processing was performed using the Agilent Mass Hunter Qualitative Analysis (version B.04.00). Putative identification of low molecular weight metabolites were established by comparing the mass spectra of the peaks with those available in the NIST mass spectral (Wiley registry NIST 11) library. The identification of peaks was based on 70% similarity index. All the GC-MS spectra were exported as.cef format, and uploaded on MPP for peak alignment, normalization, significance testing, fold change and multivariate analysis for both identified and unidentified compounds.

### Chemometric Analyses

All the available data points within full scan mode from *m/z* 50 to 650 and retention time window 6.5 to 35 minutes were used to filter the data using minimum absolute abundance of 5,000 counts. Alignment parameter was set as retention time tolerance 0.05, match factor 0.3 and delta MZ 0.2. The identified and unidentified peaks were then aligned and normalized to unit scale. A total of 735 entities were found in the entire samples after alignment. The entities obtained were filtered by frequency, p-value, fold change and CV. The one way ANOVA and Tukey’s honest significance difference (HSD) post Hoc test were applied to identify which entities were responsible for significant differences in the three groups. Different models were also generated for the significantly different metabolites.

## Results

### Significance Testing and Fold Change

Thirty one out of 735 entities among the three groups (control, pre-cancer and oral cancer) were significantly differentiated after applying filtration using frequency (appeared in more than 50% of samples in at least one group of samples), p-value < 0.05 and fold change >1.5 (Table 1). However ninteen out of thirty one entities were putatively identified (level 2 of Metabolomics Standard Initiative for the identification) by comparing the mass spectra of the peaks with those available in the NIST mass spectral library (Wiley registry NIST 11) at ≥70% similarity index, while the remaining were not identified at this similarity index. IUPAC International Chemical Identifier (InChI) for all the differentially expressed and identified metabolites is provided in Supplementary Table 2.

Tukey’s honest significance difference (HSD) post Hoc test was then applied to identify which entities were responsible for significant differences in the three groups (Table 2). It was found that twenty four metabolites were responsible for the differences among oral cancer and control group out of which fifteen were identified. Similarly, eighteen entities were distinctively expressed among pre-cancer and control. However maximum similarity was found among oral cancer and pre-cancer with eighteen entities common, eleven of them were identified.

### Clustering

**Figure 1:**
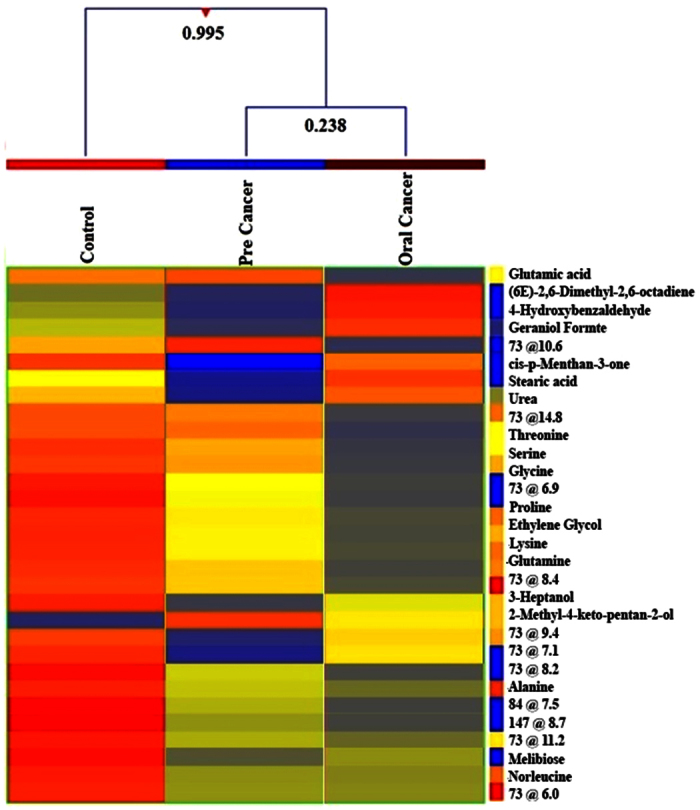
Comparison of three groups i.e., controls, pre cancer, oral cancer patients using normalized intensities of thirty one significance metabolites. The dendrogram was produced by applying a hierarchical clustering algorithm (Pearson’s centered- absolute distance metric, Complete Linkage).

Initially an unsupervised cluster analysis based on all metabolome data was performed using k-means clustering method with 500 iterations (Supplementary Figure 1). It showed no proper clustering; however color changes from healthy control to disease can be visualized. Hence for clear view, hierarchical clustering was performed by applying Pearson’s Center-Absolute, complete linkage to produce a dendrogram for clustering of sample groups using normalized intensities of thirty one significant metabolites (Fig. 1). The length of the vertical lines in the dendrogram is a measure of dissimilarity, while shorter lines demonstrate close relationship of the groups. This approach clustered the three groups into two levels. The two groups, i.e. oral cancer and pre-cancer clustered together in class I with dissimilarity level of only 0.238. In class II oral cancer, pre-cancer and control group were at dissimilarity level of 0.995. These dissimilarity levels showing that pre-cancer and oral cancer groups have close relationship while control group is the most dissimilar from other groups A heat map using all samples with normalized intensities of thirty one significant metabolites are shown in Supplementary Figure 2. From this figure it is clearly showing that control group profile is significantly different from the other two as the significantly differentiated metabolites (mostly amino acids) are comparatively higher in control group. This heat map was clustered and a dendrogram was produced by applying a hierarchical clustering algorithm (Pearson’s centered- absolute distance metric, Complete Linkage) using individual normalized intensities of thirty one significance metabolites (Supplementary Figure 3). This figure also showed maximum clustering of pre-cancerous and cancerous samples together.

### Discrimination Analysis

**Figure 2:**
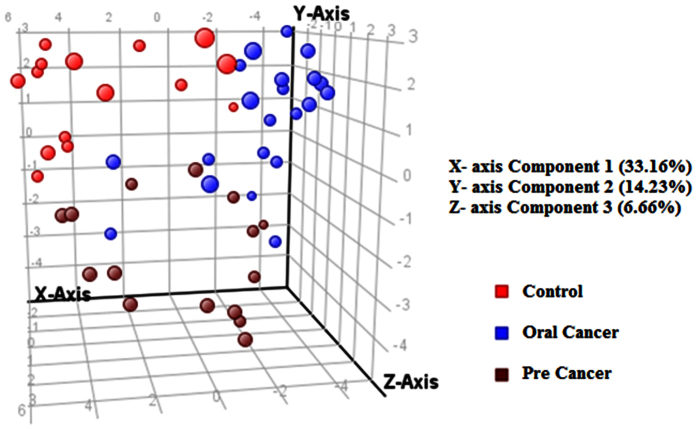
Three dimensional PCA score plot for all samples using thirty one identified and unidentified differentiative peaks.

An outlier behavior and prediction model of healthy versus disease group was built by multivariate data analysis that includes all analyzed samples on the basis of 31 metabolites. The principle component analysis (PCA) was carried out which revealed a vibrant and noteworthy difference between the non-averaged control samples and oral cancer samples. The PCA scores are shown in Fig. 2 in which each sample is denoted by a single point. The sample points of pre cancer and oral cancer was more scattered when compared to control, this could be due to the variation in the subtypes of the disease.

**Figure 3:**
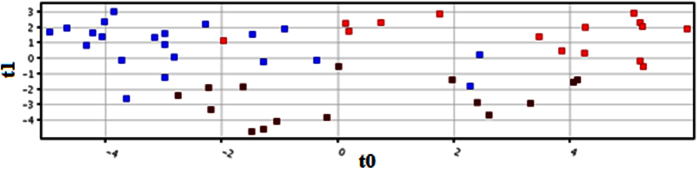
PLSDA score scatter plot discriminating among controls and oral cancer patients based on the thirty one significantly differentiate metabolite profiling data. The red, blue and brown squares indicate control, oral cancer and pre cancer patients, respectively.

Samples were classified into discrete classes also by supervised Partial Least Square Discriminant Analysis (PLSDA). Two parts of the input data were randomly assigned to the training set and remaining into the testing set. Auto-scaling was applied which involves subtracting the variable mean from each variable (data column) and dividing each by its standard deviation. This process was repeated ten times each time using a different part for testing thus using each row, once in training and testing, generating a Confusion Matrix, which gives accuracy of prediction of each class. PLS-DA score plot is shown in Fig. 3 exposing a clear separation trend between the three groups of our experiment. Sensitivity of the constructed model was calculated from the proportion of cancerous and precancerous samples that were predicted correctly and referred as true positives, while specificity was determined from the proportion of healthy control samples which were correctly predicted and these are stated as true negatives. Sensitivity and specificity of our built model was found to be 85.7% was 93.3%, respectively, while the overall accuracy of the model was 90.2% as mentioned in Supplementary Table 3. Receiver Operating Characteristics (ROC) curve was also produced for PLS-DA (Supplementary Figure 4).

### Pathway Analysis

**Figure 4:**
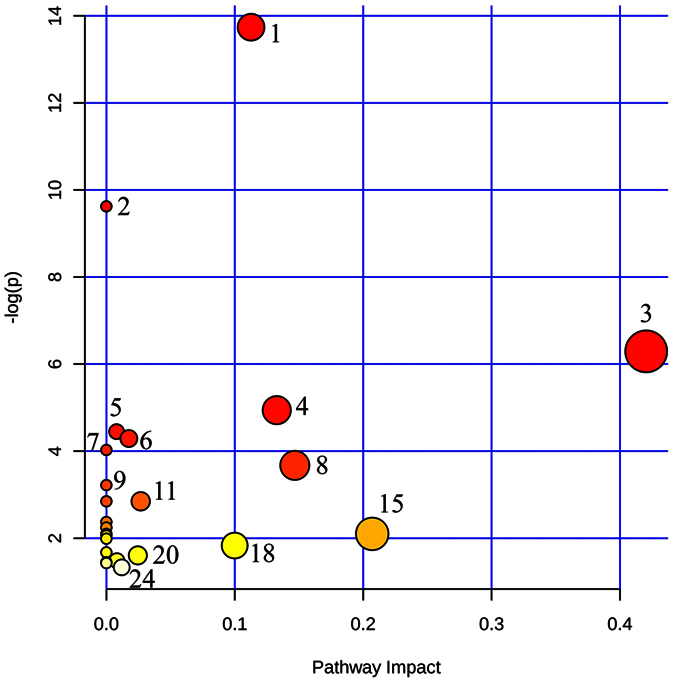
Summary of pathway analysis of metabolites found dysregulated in cancer patients as compared to healthy controls (the annotations are based on serial number given to each pathway in Table 3 and Supplementary Table 4).

MetaboAnalyst 3.0 (www.metaboanalyst.ca/) was used to identify metabolic pathways which were disturbed in diseased samples as compared to control using differentiated and identified metabolites. The summaries of pathway analysis created on the basis of hypergeometric test and relative-betweens centrality in pathway topology analysis by this program using differentiated metabolites are shown in Fig. 4. The list of discriminating identified pathways is provided in Table 3 and Supplementary Table 4. Total twenty four pathways were found to be deregulated with the highest impact of 0.42 of glycine, serine and threonine metabolic pathway. Majority of the identified pathways involved amino acids as a key metabolite in pathway.

## Discussion

Cell metabolic pathway consists of a network of proteins, interacting genes and metabolite reactions which are controlled by intricate regulatory structures. In cancer cell, deregulation in these networks results in uncontrolled growth and proliferation^15^,^16^, these metabolic alterations are the hallmark of cancer^17^.

**Figure 5:**
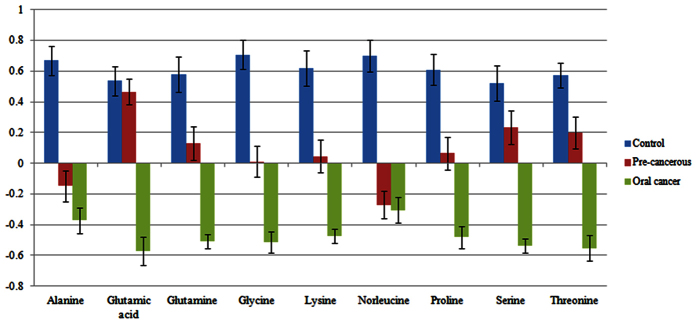
Bar graph showing normalized intensities of differentiated nine amino acids in control, pre-cancerous and oral cancer tissue samples (error bars are standard deviations in biological replicates).

Among 19 identified metabolites, 9 are amino acids that are: glycine, threonine, glutamine, lysine, proline, alanine, glutamic acid, nor leucine and serine. In the presented study, all of nine amino acids have shown very characteristic pattern of gradual decrease in relative concentration as going from healthy control tissues to pre-neoplastic lesion to oral cancer one (Fig. 5). Our results are comparable with the previous findings in which alanine, valine, lycine, glycine, threonine and glutamine level significantly decreased in other types of tumors like oral, breast, pancreatic and colorectal cancers^18^,^19^,^20^. In another study of amino acid quantification in OSF plasma samples in comparison with healthy showed reduction in the assay levels of histidine, threonine, arginine, tyrosine, isoleucine and leucine in OSF plasma^21^. However, the value for valine, phenylalanine and lysine was increased. Conversely this pattern is not common to other studies related to head and neck cancer; in which cancer tissues showed higher relative concentration of amino acids as compared to healthy tissues^12^,^22^. This anomaly suggests that for rapid cell proliferation, a secondary metabolic pathway for glucose generation is adopted which consumes glucogenic amino acids as the disease progress. In addition to this, it can also be suggested that amino acids are in continuous usage for cell proliferation which results in higher concentration at the time of supply and lower concentration after their consumption in new tumor cells production.

Cancer cells exhibit increase rate of nutrient consumption and rerouting of metabolic processes to maintain these substrate pools to favor *de novo* biosynthesis. Alteration in these metabolic pathways can be therapeutic targets for cancer research^23^. The decreased amino acid levels appear to be the result of enhanced energy metabolism or up-regulation of the related biosynthetic pathways, which are required in cell proliferation of cancer tissues. Serine and glycine are major sources of methyl groups for the one carbon pool required for a variety of biosynthetic pathways and/or DNA methylation that tumor cells use^24^. Glycine, an important intermediate in the folate metabolism, is especially altered in colon cancer^25^. Glutamine is the most abundant amino acid in plasma but in cancer patients its abundance is low as tumor cells use it for to generate energy and biosynthetic purposes^26^. It is a nitrogen donor to tumor cells, also contributes the survival of proliferating cells by maintaining mitochondrial membrane integrity, provides TCA cycle intermediates and suppresses oxidative stress by restoring glutathione to its reduced form^27^. Tumor cells consume large amounts of glutamine. Its metabolism can allow cells to meet both the anaplerotic and NADPH demands of growth^28^. Studies revealed rapid but partial glutamine oxidation and secretion of glutamine-derived carbon as lactate, establishing glutamine as an energy source in tumor cells^29^, considered a hallmark of tumor cell metabolism. Ultimately, recent advances in amino acid metabolism have revealed that targeting amino acid metabolic enzymes in cancer therapy is a promising strategy for the development of novel therapeutic agents. There are currently several drugs in clinical trials that specifically target amino acid metabolic pathways in tumor cells^24^.

Apart from amino acids, cancer cells also require fatty acids for the synthesis of membranes as well as for the generation of lipid signaling molecules to trigger cell proliferation leading to malignancy. Consistent with our results i.e., up-regulation of stearic acid in oral cancer tissue, several metabolites of the lipid metabolism pathways are detected at an elevated level in cancer cells in previous studies^30^.

Except amino acids and two alcohols, all metabolites were decreased in pre cancerous stage as compared to oral cancer. Interestingly, all significantly expressed metabolites were decreased in concentration level in precancerous tissues except one branched keto-alcohol as compared to control. These up- and down-regulations of metabolites may be due to involvement of metabolites in different metabolic pathways at the same time. For instance, TCA cycle is key metabolic pathway that unifies carbohydrate, fat, and protein metabolism. Similarly, during reduced supply of sugars, a complementary pathway for the production of glucose may be regulated at the cost of glucogenic amino acids and lipids. As in this study, a fatty acid, stearic acid, is found to be down regulated in precancerous and up-regulated in cancer stage. The catabolic product of free fatty acid enters into the kreb’s cycle and the intermediates of this cycle further generate few non-essential amino acids like glutamic acid in our study. However, high level of fatty acid in oral cancer may be due to demand of rapid cell proliferation.

## Conclusion

Our study has shown that a GC-MS-based metabolite profiling and extensive chemometric analysis of tissue is able to identify biomarker metabolites which can significantly differentiate oral cancer from the control groups. Identification of unknown metabolites with high resolution can increase human metabolome and ultimately help in early diagnostic biomarker identification of oral cancer. In this study, oral cancer and pre-cancerous sample showed a decreased level of amino acids compared to control. Such measurements propose that modulation of amino acid metabolism may represent new potential and novel strategy for the treatment of premalignant and malignant oral lesions. However, further studies are needed to elucidate the potential of these profiles in the pathogenesis of OSF and OSCC; and its implications in the malignant transformation potential of such condition.

## Additional Information

**How to cite this article**: Musharraf, S. G. *et al*. Metabolite Profiling of Preneoplastic and Neoplastic Lesions of Oral Cavity Tissue Samples Revealed a Biomarker Pattern. *Sci. Rep.* **6**, 38985; doi: 10.1038/srep38985 (2016).

**Publisher’s note:** Springer Nature remains neutral with regard to jurisdictional claims in published maps and institutional affiliations.

## Supplementary Material

Supplementary Information

## Figures and Tables

**Table 1:** List of differentiative metabolites (31 entities) among oral cancer, pre-cancer and control groups at p < 0.05 and fold change >1.5.

S. No.	(a) Compounds or (b) Base peak (*m/z*)	Retention Time (min)	*p* (Corr)	Log FC (Oral Cancer vs Control)	Log FC (Pre Cancer vs Control)	Log FC (Pre Cancer vs Oral Cancer)
1.	(6E)-2,6-Dimethyl-2,6-octadiene^a^	10.699	1.03 × 10^−04^	1.062668	−0.28537	−1.3480332
2.	2-Methyl-4-keto-pentan-2-ol^a^	6.422	3.32 × 10^−03^	0.724624	1.22061	0.49598637
3.	3-Heptanol^a^	6.450	3.32 × 10^−03^	−0.74727	−1.21632	−0.46905234
4.	4-Hydroxybenzaldehyde^a^	11.100	3.18 × 10^−04^	0.924649	−0.39752	−1.3221662
5.	Alanine^a^	7.314	5.02 × 10^−03^	−1.04076	−0.81737	0.22338897
6.	cis-*p*-Menthan-3-one^a^	8.139	6.43 × 10^−04^	0.514114	−0.79117	−1.3052865
7.	Ethylene Glycol^a^	8.199	3.35 × 10^−03^	−1.12252	−0.5831	0.5394201
8.	Geraniol Formte^a^	10.071	2.05 × 10^−03^	0.726486	−0.43886	−1.165349
9.	Glutamic acid^a^	14.196	2.54 × 10^−03^	−0.90651	−0.129154	1.0356688
10.	Glutamine^a^	15.899	3.79 × 10^−03^	−1.08571	−0.44992	0.6357883
11.	Glycine^a^	7.500	1.69 × 10^−03^	−1.22106	−0.69551	0.52554303
12.	Lysine^a^	17.438	3.83 × 10^−03^	−1.09429	−0.57469	0.51960033
13.	Melibiose^a^	24.133	6.43 × 10^−04^	−1.10813	−1.29065	−0.18252334
14.	Norleucine^a^	9.699	4.01 × 10^−03^	−1.00534	−0.97022	0.035111338
15.	Proline^a^	10.100	3.83 × 10^−03^	−1.09033	−0.54376	0.5465712
16.	Serine^a^	10.899	3.35 × 10^−03^	−1.05951	−0.28753	0.77197886
17.	Stearic acid^a^	20.298	2.38 × 10^−03^	0.24771	−0.90751	−1.1552157
18.	Threonine^a^	11.300	2.45 × 10^−03^	−1.12508	−0.37226	0.75282097
19.	Urea^a^	9.211	3.83 × 10^−03^	−0.98554	−0.14505	0.8404852
20.	73^b^	6.000	6.89 × 10^−03^	−0.94928	−0.91998	0.029298544
21.	73^b^	6.900	6.43 × 10^−04^	−1.30969	−0.82124	0.48845243
22.	73^b^	7.100	1.20 × 10^−03^	−0.53524	−1.34792	−0.8126821
23.	73^b^	8.245	4.25 × 10^−04^	−1.34824	−0.95951	0.38872778
24.	73^b^	8.399	6.43 × 10^−03^	−1.01674	−0.41896	0.5977822
25.	73^b^	9.399	3.57 × 10^−03^	−0.4036	−1.18666	−0.78305763
26.	73^b^	10.399	6.86 × 10^−06^	−0.16779	−1.58506	−1.4172763
27.	73^b^	10.600	9.41 × 10^−04^	−0.79637	0.435748	1.2321197
28.	73^b^	11.199	2.79 × 10^−03^	−1.11279	−0.92984	0.1829573
29.	73^b^	14.799	2.45 × 10^−03^	−1.01745	−0.07408	0.94337636
30.	84^b^	7.590	1.03 × 10^−04^	−1.44077	−1.117	0.32377183
31.	147^b^	8.699	3.56 × 10^−05^	−1.48922	−1.24458	0.24464041

FC = fold change.

^a^Identified metabolites.

^b^Unidentified metabolites.

**Table 2:** Matrix produced after Tukey’s honest significance difference (HSD) post Hoc test: number of entities responsible for significant differences between groups shown in upper half matrix while non-significant entities are in lower half matrix.

Group Name	Pre Cancer	Oral Cancer	Control
Pre Cancer	31	15	18
Oral Cancer	18	31	24
Control	15	9	31

**Table 3:** List of significant dysregulated pathways in cancer and pre-cancerous patients in comparison to healthy controls with threshold of <0.05 FDR.

Sr.#	Pathway Name	Total Compound Present in Pathway	Hits	Raw p	−log (p)	Holm p	FDR	Impact
1.	Aminoacyl-tRNA biosynthesis	75	6	1.08E-06	13.737	8.65E-05	8.65E-05	0.11268
2.	Cyanoamino acid metabolism	16	3	6.62E-05	9.6221	0.00523	0.00265	0
3.	Glycine, serine and threonine metabolism	48	3	0.0018508	6.2922	0.14436	0.04935	0.42039
