# Supplementary material for: Metabolite Profiling of Preneoplastic and Neoplastic Lesions of Oral Cavity Tissue Samples Revealed a Biomarker Pattern
Source: Sci Rep. 2016 Dec 13;6:38985. doi: 10.1038/srep38985 (PMC5154177; doi:10.1038/srep38985)
Supplement: Supplementary Information [file srep38985-s1.pdf]

Metabolite Profiling of Preneoplastic and Neoplastic Lesions of Oral Cavity Tissue Samples Revealed a Biomarker Pattern

**Syed Ghulam Musharraf* [1,2], *Najia Shahid* [2], *Syed Muhammad Ali Naqvi*[3], *Mahwish Saleem*[1], *Amna Jabbar Siddiqui* [2], *and Anwar Ali*[3]

[1]Dr. Panjwani Center for Molecular Medicine and Drug Research, International Center for Chemical and Biological Sciences, University of Karachi, Karachi-75270, Pakistan.
[2]H.E.J. Research Institute of Chemistry, International Center for Chemical and Biological Sciences, University of Karachi, Karachi-75270, Pakistan.
[3]Dr. Ishrat-ul-Ebad Institute of Oral Health Sciences, DOW University of Health Sciences, Karachi-74200, Pakistan.

*Corresponding author Tel.: +92 021-34824924; 4819010; fax: +92 021-34819018-9
Email: musharraf1977@yahoo.com,

Supplementary table 1: Subject characteristics

**Groups**	**Control**	**Pre-cancerous**	**Oral cancer**
Age in years			
Min-Max (median)	20-65 (30.5)	19-60 (35)	15-62 (43.5)
Gender			
Male	10	10	19
Female	5	5	2

Supplementary table 2: IUPAC International Chemical Identifier (InChI) for all the differentially expressed metabolites among controls and patients.

Identified metabolite	International Chemical Identifier
(6E)-2,6-Dimethyl-2,6-octadiene	1S/C10H18/c1-5-10(4)8-6-7-9(2)3/h5,7H,6,8H2,1-4H3/b10-5+
2-Methyl-4-keto-pentan-2-ol	1S/C6H12O2/c1-5(7)4-6(2,3)8/h8H,4H2,1-3H3
Ethylene Glycol	1S/C2H6O2/c3-1-2-4/h3-4H,1-2H2
Glutamic acid	1S/C5H9NO4/c6-3(5(9)10)1-2-4(7)8/h3H,1-2,6H2,(H,7,8)(H,9,10)/t3-/m0/s1
4-Hydroxybenzaldehyde	1S/C7H6O2/c8-5-6-1-3-7(9)4-2-6/h1-5,9H
cis-*p*-Menthan-3-one	1S/C10H18O/c1-7(2)9-5-4-8(3)6-10(9)11/h7-9H,4-6H2,1-3H3
Melibiose	1S/C12H22O11/c13-1-3-5(14)8(17)10(19)12(23-3)21-2-4-6(15)7(16)9(18)11(20)22-4/h3-20H,1-2H2/t3-,4-,5+,6-,7+,8+,9-,10-,11-,12+/m1/s1
Geraniol Formte	1S/C11H18O2/c1-10(2)5-4-6-11(3)7-8-13-9-12/h5,7,9H,4,6,8H2,1-3H3/b11-7+
Glycine	1S/C2H5NO2/c3-1-2(4)5/h1,3H2,(H,4,5)
Lysine	1S/C6H14N2O2/c7-4-2-1-3-5(8)6(9)10/h5H,1-4,7-8H2,(H,9,10)/t5-/m0/s1
Norleucine	1S/C6H13NO2/c1-2-3-4-5(7)6(8)9/h5H,2-4,7H2,1H3,(H,8,9)/t5-/m0/s1
Proline	1S/C5H9NO2/c7-5(8)4-2-1-3-6-4/h4,6H,1-3H2,(H,7,8)/t4-/m0/s1
Threonine	1S/C4H9NO3/c1-2(6)3(5)4(7)8/h2-3,6H,5H2,1H3,(H,7,8)/t2-,3+/m1/s1
Stearic acid	1S/C18H36O2/c1-2-3-4-5-6-7-8-9-10-11-12-13-14-15-16-17-18(19)20/h2-17H2,1H3,(H,19,20)
Serine	1S/C3H7NO3/c4-2(1-5)3(6)7/h2,5H,1,4H2,(H,6,7)
3-Heptanol	1S/C7H16O/c1-3-5-6-7(8)4-2/h7-8H,3-6H2,1-2H3
Urea	1S/CH4N2O/c2-1(3)4/h(H4,2,3,4)
Alanine	1S/C3H7NO2/c1-2(4)3(5)6/h2H,4H2,1H3,(H,5,6)/t2-/m0/s1
Glutamine	1S/C5H10N2O3/c6-3(5(9)10)1-2-4(7)8/h3H,1-2,6H2,(H2,7,8)(H,9,10)/t3-/m0/s1

Supplementary table 3: Matrix produced after PLSDA model generation using randomly selected samples.

	Control Predicted	Pre-cancerous Predicted	Oral cancer Predicted	Accuracy
True Control	14	1	0	93.3
True Pre-cancerous	1	12	3	80.0
True Oral cancer	0	1	20	95.5
Overall Accuracy				90.2

Supplementary table 4: List of dysregulated pathways in cancer and pre-cancerous patients in comparison to healthy controls with threshold of >0.05 FDR (Sr # is continued from table 3.

Sr.#	Pathway Name	Total Compound Present in Pathway	Hits	Raw p	-log(p)	Holm p	FDR	Impact
4.	Arginine and proline metabolism	77	3	0.0071449	4.9414	0.55015	0.1429	0.13267
5.	Purine metabolism	92	3	0.011699	4.4482	0.88914	0.18275	0.00794
6.	Methane metabolism	34	2	0.013706	4.2899	1	0.18275	0.01751
7.	Nitrogen metabolism	39	2	0.017831	4.0268	1	0.20378	0
8.	Lysine degradation	47	2	0.025386	3.6735	1	0.25386	0.14675
9.	Pyrimidine metabolism	60	2	0.039956	3.22	1	0.35517	0
10.	Biotin metabolism	11	1	0.057949	2.8482	1	0.42144	0
11.	D-Glutamine and D-glutamate metabolism	11	1	0.057949	2.8482	1	0.42144	0.02674
12.	Sulfur metabolism	18	1	0.093194	2.3731	1	0.62129	0
13.	Porphyrin and chlorophyll metabolism	104	2	0.10581	2.2461	1	0.63609	0
14.	Thiamine metabolism	24	1	0.12243	2.1002	1	0.63609	0
15.	Alanine, aspartate and glutamate metabolism	24	1	0.12243	2.1002	1	0.63609	0.20703
16.	Sphingolipid metabolism	25	1	0.12722	2.0618	1	0.63609	0
17.	Valine, leucine and isoleucine biosynthesis	27	1	0.13672	1.9898	1	0.64339	0
18.	Lysine biosynthesis	32	1	0.16006	1.8322	1	0.71138	0.09993
19.	Glutathione metabolism	38	1	0.1873	1.675	1	0.78863	0
20.	Galactose metabolism	41	1	0.20061	1.6064	1	0.80245	0.02448
21.	Primary bile acid biosynthesis	47	1	0.22663	1.4844	1	0.83252	0.00822
22.	Fatty acid biosynthesis	49	1	0.23513	1.4476	1	0.83252	0
23.	Glyoxylate and dicarboxylate metabolism	50	1	0.23935	1.4298	1	0.83252	0
24.	Cysteine and methionine metabolism	56	1	0.2642	1.331	1	0.88067	0.01197

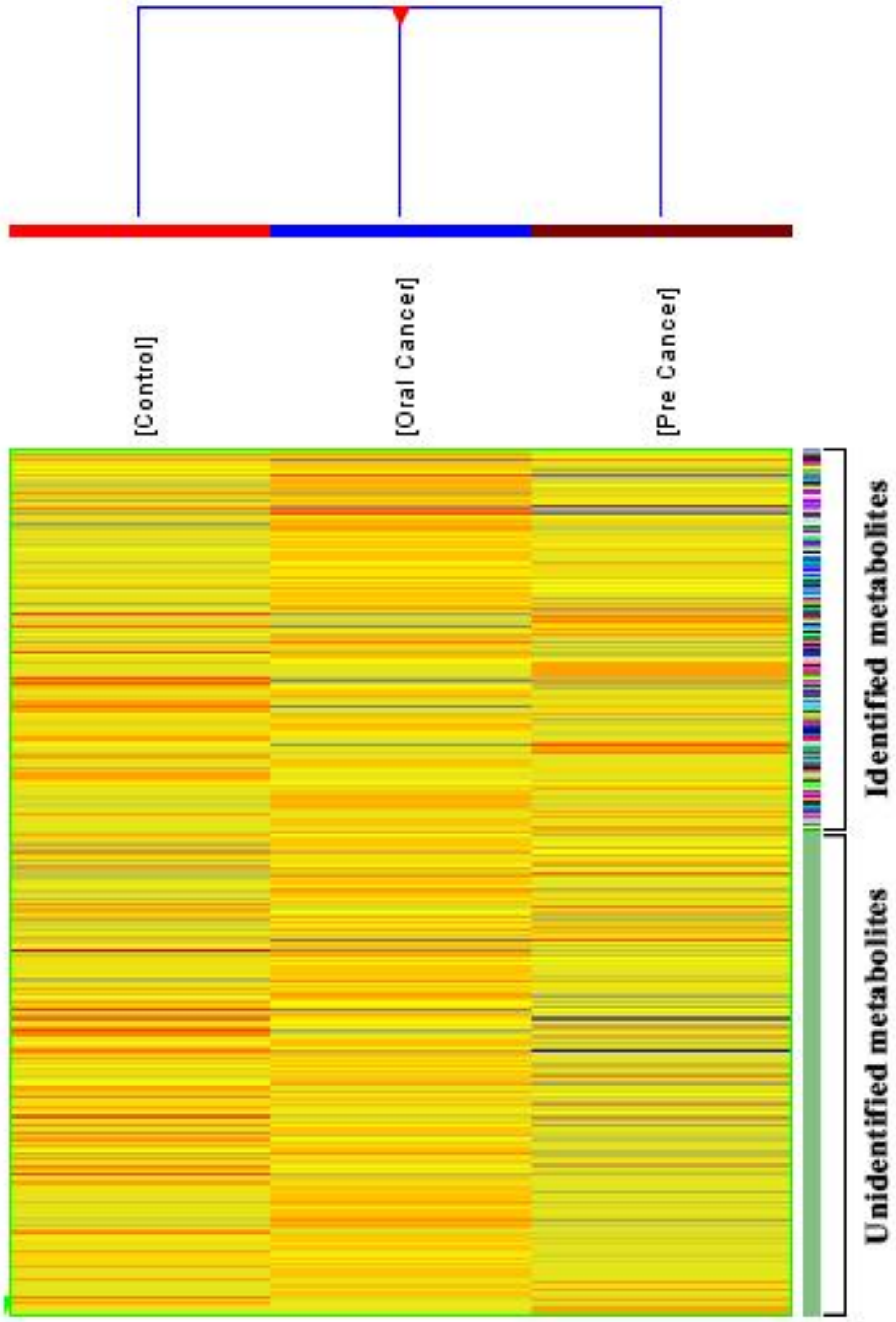

Supplementary Figure 1 Comparison of three groups i.e., controls, pre cancer, oral cancer patients using average normalized intensities of all metabolome. The dendrogram was produced by applying a k-means clustering algorithm (Pearson's centered- absolute distance metric).

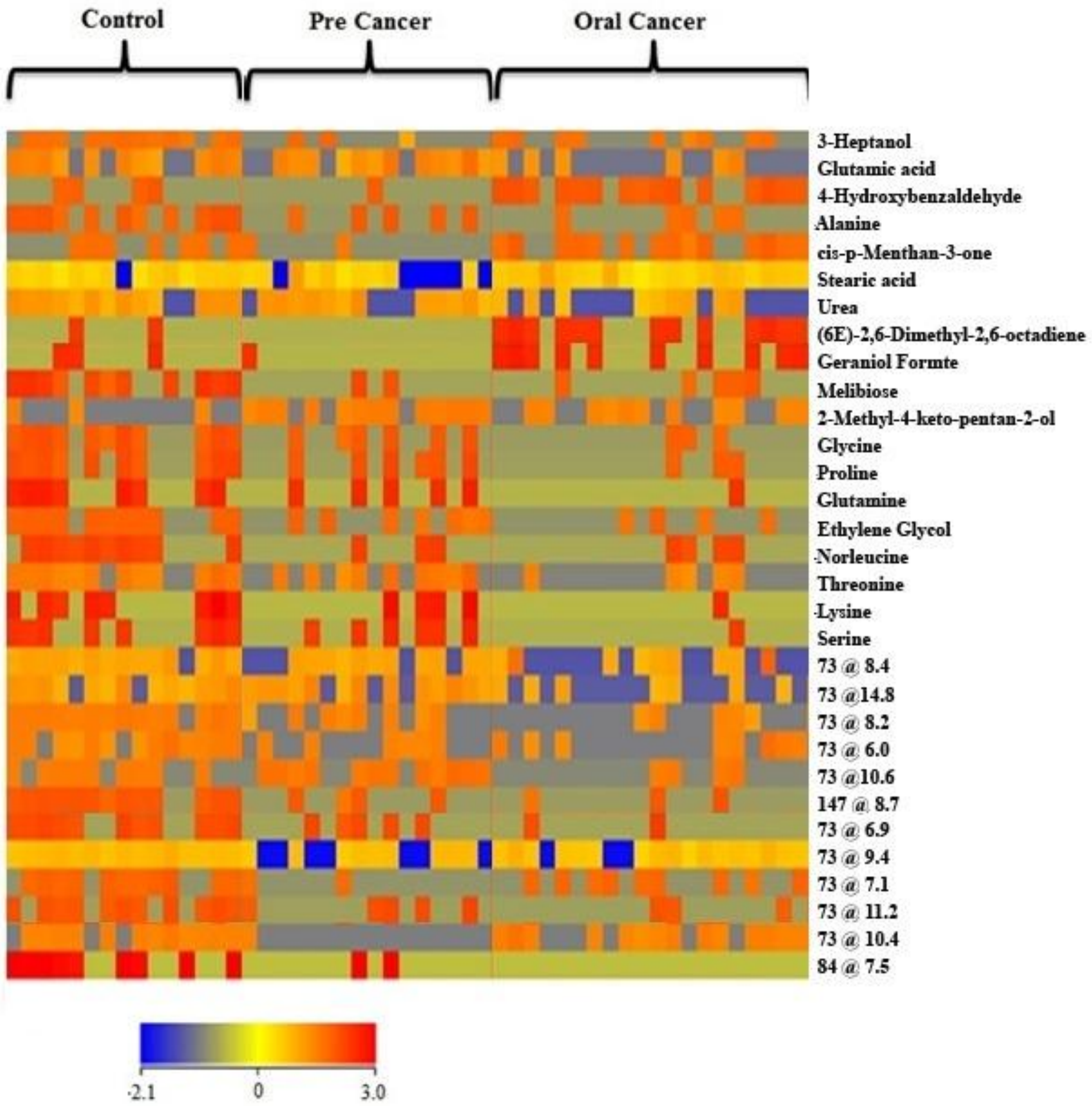

Supplementary Figure 2 Heat map of samples showing normalized intensities of thirty one statistically significant metabolites. Identified compounds are labeled by their names while unidentified compounds are labeled by their base peaks (*m/z*) @ retention time (RT) in minutes.

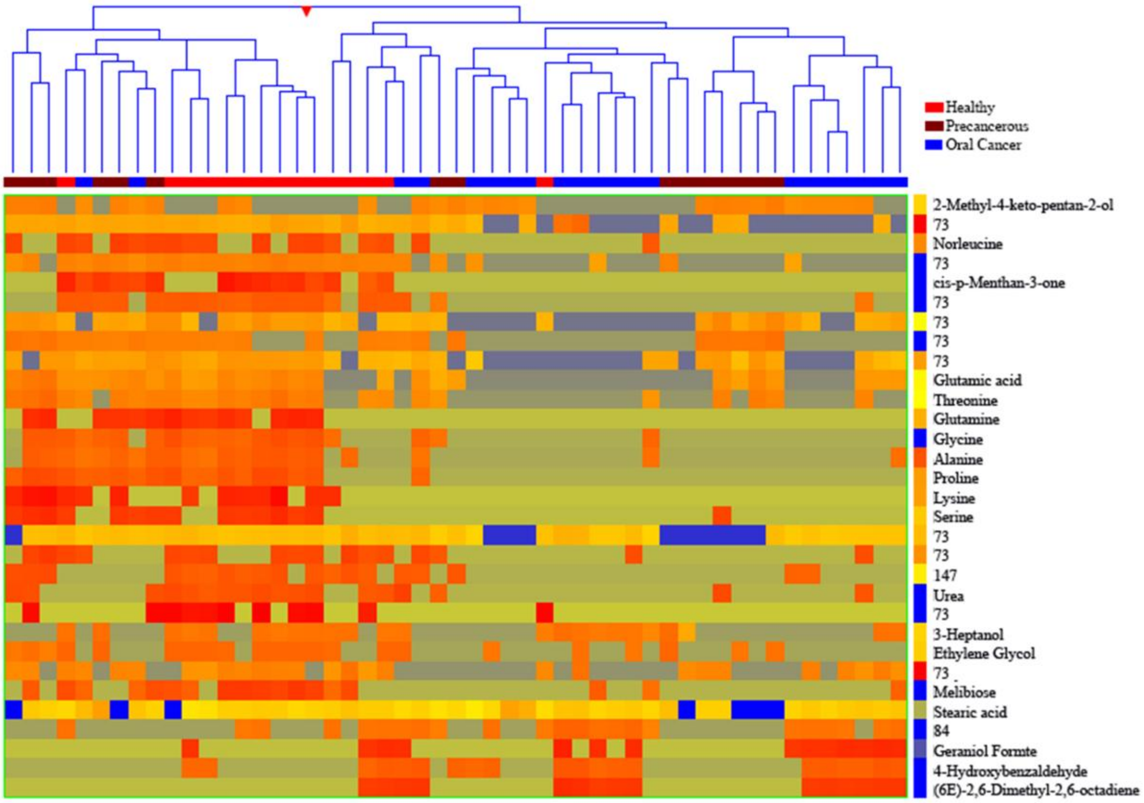

Supplementary Figure 3 Comparison of three groups i.e., controls, pre cancer, oral cancer patients using individual normalized intensities of thirty one significance metabolites. The dendrogram was produced by applying a hierarchical clustering algorithm (Pearson's centered- absolute distance metric, Complete Linkage).

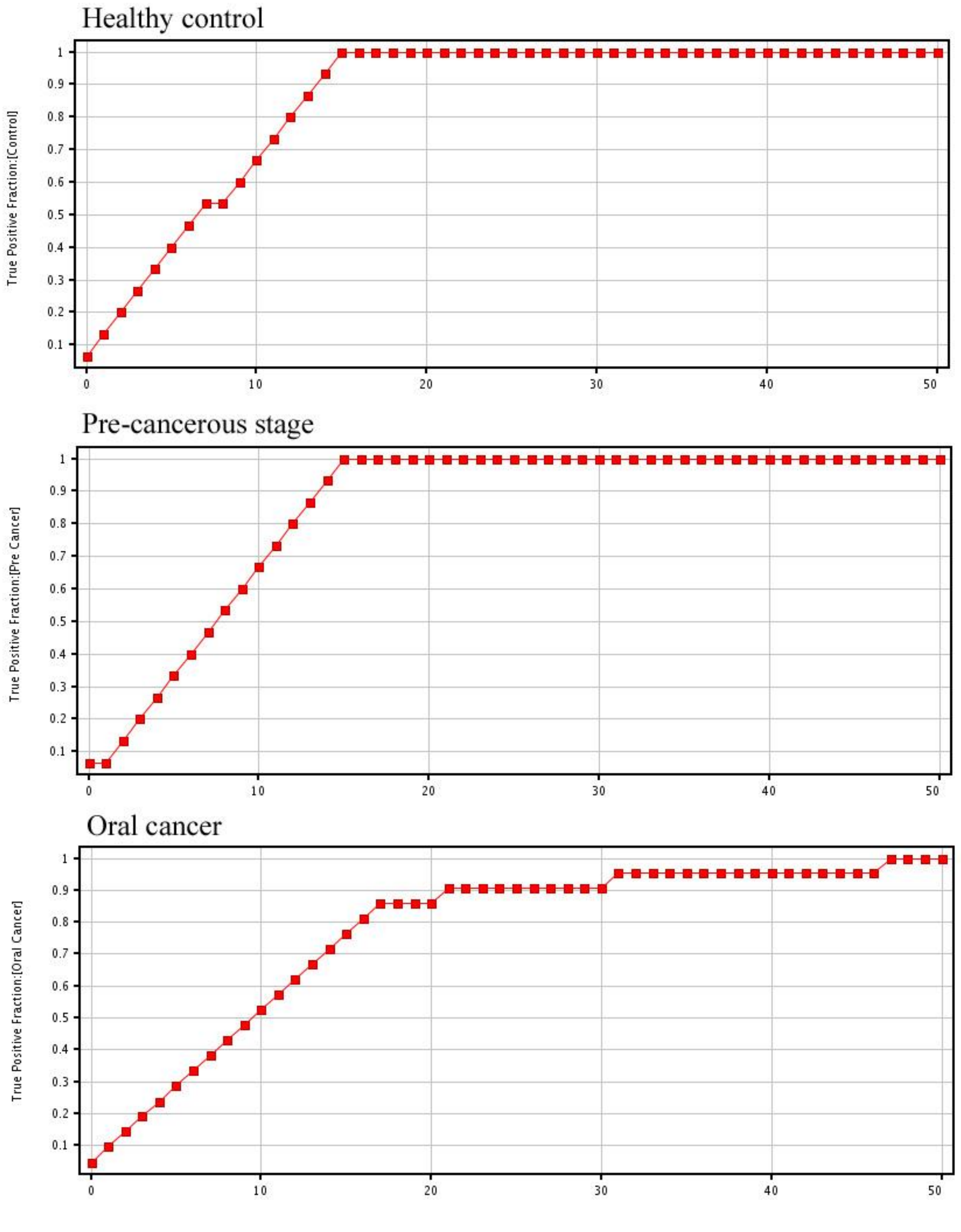

Supplementary Figure 4: Reciever operating characteristic (ROC) plot for PLS-DA model showing sensitivity on y-axis and 1-specificity on x-axis.
